# Molecular biomarkers of leukemia: convergence-based drug resistance mechanisms in chronic myeloid leukemia and myeloproliferative neoplasms

**DOI:** 10.3389/fphar.2024.1422565

**Published:** 2024-07-22

**Authors:** Meike Kaehler, Nikolas von Bubnoff, Ingolf Cascorbi, Sivahari Prasad Gorantla

**Affiliations:** ^1^ Institute of Experimental and Clinical Pharmacology, University Hospital Schleswig-Holstein, Kiel, Germany; ^2^ Department of Hematology and Oncology, University Medical Center Schleswig-Holstein, Lübeck, Germany

**Keywords:** chronic myeloid leukemia (CML), myeloprolifarative neoplasms, drug resistance, tyrosine kinase inhibitors (TKI), BCR::ABL1, JAK2

## Abstract

Leukemia represents a diverse group of hematopoietic neoplasms that can be classified into different subtypes based on the molecular aberration in the affected cell population. Identification of these molecular classification is required to identify specific targeted therapeutic approaches for each leukemic subtype. In general, targeted therapy approaches achieve good responses in some leukemia subgroups, however, resistance against these targeted therapies is common. In this review, we summarize molecular drug resistance biomarkers in targeted therapies in BCR::ABL1-driven chronic myeloid leukemia (CML) and JAK2-driven myeloproliferative neoplasms (MPNs). While acquisition of secondary mutations in the BCR::ABL1 kinase domain is the a common mechanism associated with TKI resistance in CML, in JAK2-driven MPNs secondary mutations in JAK2 are rare. Due to high prevalence and lack of specific therapy approaches in MPNs compared to CML, identification of crucial pathways leading to inhibitor persistence in MPN model is utterly important. In this review, we focus on different alternative signaling pathways activated in both, BCR::ABL1-mediated CML and JAK2-mediated MPNs, by combining data from *in vitro* and in vivo-studies that could be used as potential biomarkers of drug resistance. In a nutshell, some common similarities, especially activation of PDGFR, Ras, PI3K/Akt signaling pathways, have been demonstrated in both leukemias. In addition, induction of the nucleoprotein YBX1 was shown to be involved in TKI-resistant JAK2-mediated MPN, as well as TKI-resistant CML highlighting deubiquitinating enzymes as potential biomarkers of TKI resistance. Taken together, whole exome sequencing of cell-based or patients-derived samples are highly beneficial to define specific resistance markers. Additionally, this might be helpful for the development of novel diagnostic tools, e.g., liquid biopsy, and novel therapeutic agents, which could be used to overcome TKI resistance in molecularly distinct leukemia subtypes.

## Introduction

Among all cancers, leukemic malignancies derived from immature or mature blood cells make up to 3% of newly diagnosed cases, with the majority of cases in children (Siegel et al., 2023). Within leukemia, myeloid cancers are a heterogenous group of several neoplasms with variable therapy options and survival rates for the patients. Myeloproliferative neoplasms (MPNs) are classified as different entities based on the molecular alteration present in effected cell population: Polycythemia vera (PV), essential thrombocythemia (ET), primary myelofibrosis (PMF), chronic neutrophilic leukemia (CNL), chronic eosinophilic leukemia (CEL), juvenile myelomonocytic leukemia (JMML) and chronic myeloid leukemia (CML, Table 1). Despite an overall survival rate of 66.7% for myeloproliferative cancers, the 10-year overall survival rates for myeloid neoplasms vary with 68% for ET, 64% for PV and 21% for PMF and 80% for CML (Hultcrantz et al., 2012; Szuber et al., 2019; Hochhaus et al., 2020). In all cancer entities, there is an urgent need and optimize treatment options to identify patients suitable for therapy, at risk for relapses and to find alternative treatment regimens for distinct patient groups. Here, we review on potential biomarkers on drug resistance, which could be used in TKI-resistant CML and MPNs.

**TABLE 1:** Overview of myeloid neoplasms with their respective hallmarks, drugs for treatment, mechanisms of resistance and recommended drugs to overcome resistance.

Neoplasm	Hallmarks	Drugs	Mechanisms of resistance	Recommended drugs to overcome resistance	References
BCR::ABL1-dependent					
Chronic myeloid leukemia (CML)	*BCR::ABL1* fusion gene	imatinib nilotinib, dasatinib, bosutinib ponatinib asciminib	BCR::ABL1 mutations (especially p.T315I) Deregulation of ADME genes (metabolism, transport) Activation of alternative signaling pathways: *ASXL1*, *DMNT3A*, *SETBP1*, miR-203 *KRAS*, *NRAS*, *PTPN11, YBX1* (*in vitro*) Markers of sustained MMR: *BCR::ABL1* transcript ratio, duration of MMR	TKI switch, novel BCR::ABL1 inhibitors More potent TKI Non-BCR::ABL1 inhibitors	Eiring and Deininger 2014, Braun et al., 2020, Alves et al., 2021 Peng et al., 2005 Kaehler and Cascorbi 2023, Kumar et al., 2022, Meenakshi Sundaram et al., 2019 Minciacchi et al., 2021 Kim et al., 2017 Shibuta et al., 2013 Kaehler et al., 2023 Lei et al., 2021, Huang et al., 2016; Meenakshi Sundaram et al., 2019; Alves et al., 2021; Costa et al., 2024 Campiotti et al., 2017, Han 2023
BCR::ABL1-independent					
Polycythemia vera (PV) Essential thrombocythemia (ET) Primary myelofibrosis (PMF)	*JAK2-*p.V617F *JAK2-*p.V617F, calreticulin *JAK2* p.V617F, calreticulin and MPL	ruxolitinib fedratinib	*JAK2* mutations PDGFRA activation *RAS* mutations Overexpression of YBX1 Inactivation of BAD Cytokine overexpression (mainly IL-6, TNFα, IFNγ)	Type II JAK2 inhibitors BCL-2 inhibitors MEK/ERK inhibitors HSP90 inhibitors	Koppikar et al., 2012, Tvorogov et al., 2018, Deshpande et al., 2021, Gorantla et al., 2024, Meyer et al., 2015; Nair et al., 2023, Brkic et al., 2021, Bhagwat et al., 2014 Stivala et al. (2019) Mylonas et al. (2020) Jayavelu et al. (2020) Winter et al., 2014 Fisher et al. (2019)
Chronic neutrophilic leukemia (CNL)	*CSF3R*	ruxolitinib	Mutations in *SETBP1* *GATA* and *KIT*		

### BCR::ABL1-driven myeloproliferative neoplasms: CML

**FIGURE 1:**
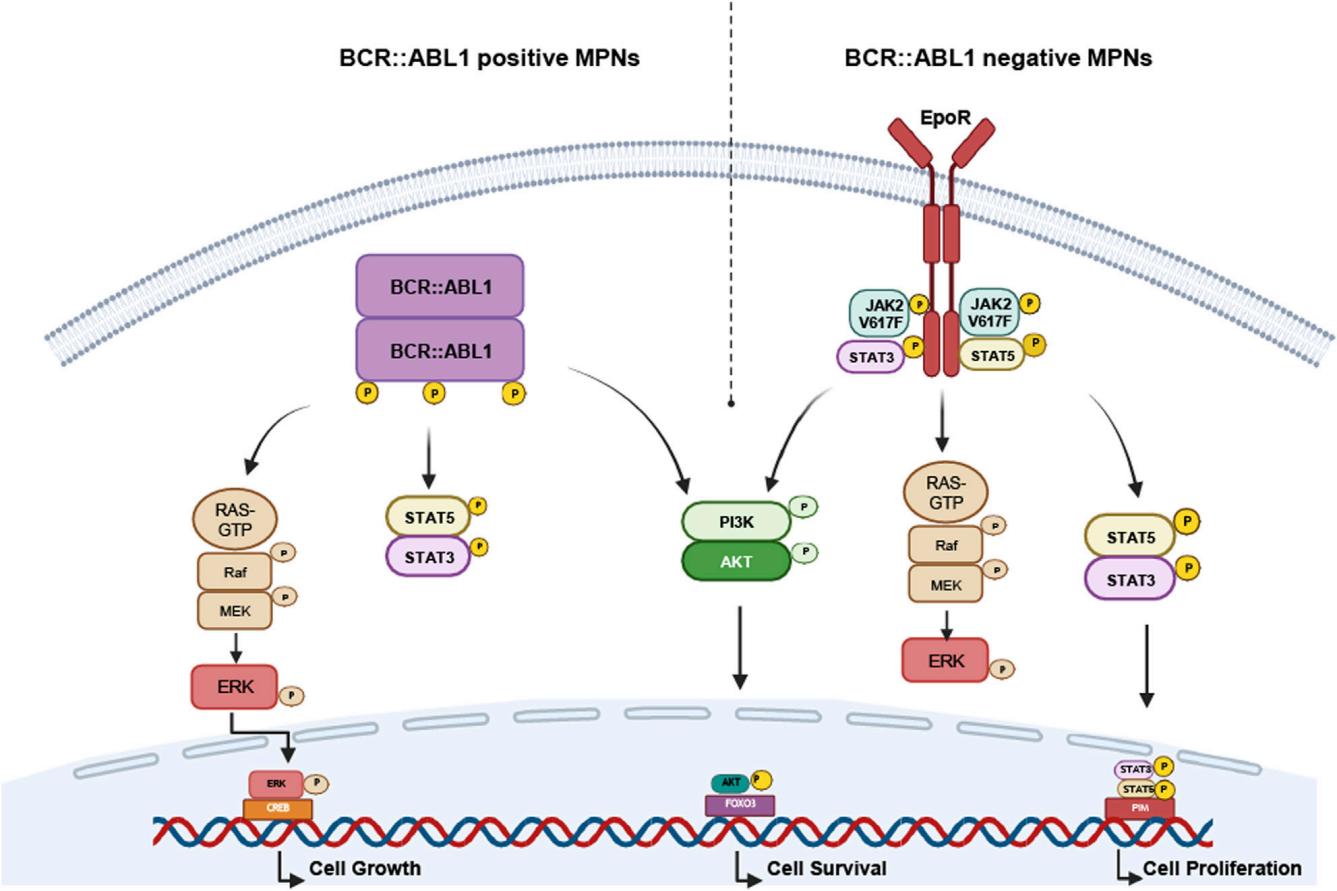
Oncogenic mechanisms of BCR::ABL1-positive and -negative myeloproliferative neoplasms (MPNs). Schematic representation of the oncogenic mechanisms underlying BCR::ABL1-dependent neoplasms (esp. CML) and -negative neoplasms (PV, ET, PMF, CNL). The presence and constitutive activation of the oncogene leads to permanent activation of the Ras/MAP, JAK/STAT or PI3K/Akt signaling pathway resulting in cell growth, proliferation and survival and thus, malignant progression. CML: chronic myeloid leukemia, CNL: chronic neutrophilic leukemia, ET: essential thrombocythemia, PMF: primary myelofibrosis. PV: polycythemia vera, EpoR: erythropoietin receptor.

CML is a rare hematopoietic neoplasm with an incidence of 1:100.000 contributing to 20%–25% of adult leukemia (Hochhaus et al., 2017). Being predominantly caused by reciprocal translocation t (9; 22) (q34; q11) forming the so-called Philadelphia chromosome (Ph), the emerging fusion oncogene *BCR::ABL1* is the hallmark of the disease (Nowell and Hungerford, 1960; Rowley, 1973; Heisterkamp et al., 1983) (Figure 1). It is detectable in 95% of all CML cases, but also in 20% of Ph-positive acute lymphoblastic leukemia (ALL) (Radich, 2001; Soverini et al., 2014). While the function of the phosphoprotein encoded by *BCR* (breakpoint cluster region protein) is widely unknown, *ABL1* is a cytosolic Abelson tyrosine kinase involved in various signaling pathways, such as Ras-MAP-kinase, JAK/STAT and PI3K/Akt (McCubrey et al., 2008). Thus, the occurrence of the BCR::ABL1 kinase leads to constitutive activation of these signaling pathways resulting in malignant transformation. As CML is a chronic disease, it slowly progresses as an initial perennial chronic phase to an accelerated phase and eventually, if untreated, ends in a terminal blast crisis accompanied with an increase in (severe) symptoms, such as splenomegaly, abdominal pain, pathologic left shift and accumulation of immature blood cells (Savage et al., 1997; Sawyers, 1999). Alternatively, the WHO Classification adopted a biphasic scheme, which only includes the chronic phase and blast crisis, while the accelerated phase is considered as high-risk chronic phase (Khoury et al., 2022).

Since the development of tyrosine kinase inhibitors (TKIs) targeting the disease-causing BCR::ABL1 kinase, CML can be effectively treated. These TKIs mainly bind to the BCR::ABL1 kinase domain and prevent phosphorylation of downstream targets resulting in growth inhibition and eventually, cell death. The use of TKIs, especially the 2-phenylpyrimidine imatinib, in CML has been tremendously successful with an overall 10-year survival of more than 80% of patients (Druker et al., 1996; Hochhaus et al., 2017). Thus, this success in rational drug designed therapy gave rise to the development of the whole field of small molecules in targeted therapy regimens. Although the success of TKIs in CML is tremendous, 20%–25% of CML patients undergoing TKI therapy develop resistances within 5-years of treatment resulting in relapses (Milojkovic and Apperley, 2009; Hochhaus et al., 2017) resulting in the development of novel BCR::ABL1 TKIs. Meanwhile, there is a variety of BCR:ABL1 TKIs used for CML, namely, the second generation nilotinib, dasatinib, bosutinib, third generation ponatinib, and the novel STAMP-inhibitor (specifically targeting the BCR:ABL1 myristoyl pocket) asciminib (Schoepfer et al., 2018; Luciano et al., 2020), which all differ in the spectrum of targeted kinases and side effects (Kaehler and Cascorbi, 2023) (Table 1).

### Potential biomarkers in CML treatment

About a quarter of TKI-treated patients develop TKI resistance within 5 years of treatment duration, while others achieve a long-lasting deep molecular response. This raises the question of putative biomarkers that can be used to identify potential TKI non-responders or patients suffering from relapses, but also potential candidates for a TKI discontinuation.

### Life-long therapy: Yes or no?

To this date, the use of TKIs in CML is considered as a life-long therapy, as the predominant effect of TKIs is the inhibition of cell growth by prevention of downstream signaling. However, eventually, the cells undergo apoptosis. The success of TKI therapy can be monitored by remission rates on different physiological levels: Hematological remission, the normalization of the blood cell count of especially leukocytes, cytogenetic remission, the absence of the Ph-chromosome and molecular response, the number of BCR::ABL1 transcripts (O'Hare et al., 2012; Baccarani et al., 2013). A *BCR::ABL1* transcript <0.1% IS is considered as deep molecular response. Besides these laboratory parameters, the SOKAL score is levied to evaluate the patient risk based on physiological factors, such as spleen size or blast count (O'Hare et al., 2012). However, this score was developed in the pre-TKI era and is replaced by the European Treatment and Outcome Study (EUTOS) long-term survival (ELTS). The latter considers the age, spleen size, the count of peripheral blasts and thrombocytopenia under imatinib therapy (Sato et al., 2020). As a huge number of patients are in perennial deep molecular response and the life expectancy of CML patients is similar to the general population (Hochhaus et al., 2020), the question occurred if the TKI treatment can be safely terminated without a risk of relapses. Overall, the outcome of discontinuation studies is quite variable, as a meta-analysis including 14 studies with 2,040 patients showed median relapse rates between 38% and 64% (Campiotti et al., 2017; Han, 2023). Further, in a study from Etienne *et al.*, molecular recurrence after imatinib discontinuation was analyzed with an overall incidence of 60%. Interestingly, the huge majority of patients suffer from relapses within 12 months after discontinuation (Etienne et al., 2017). Moreover, the probability for maintenance of treatment-free survival after dasatinib discontinuation was 48% after 12 months (Shah et al., 2023). Therefore, the challenge remains to identify suitable CML patients for TKI discontinuation. As potential biomarkers, the disease status (chronic phase), the *BCR::ABL1* fusion transcript (e13a2 or e14a2), SOKAL score, therapy duration (>5 years) and duration of a deep molecular response with a *BCR::ABL1* transcript <10^−4^–10^−5^ are considered (Han, 2023) (Table 1). To this date, the treatment recommendation still remains a life-long therapy. However, this might be changed as soon as better parameters are evaluated to identify eligible patients for TKI discontinuation.

### *BCR::ABL1* mutations: prediction of TKI response

**FIGURE 2:**
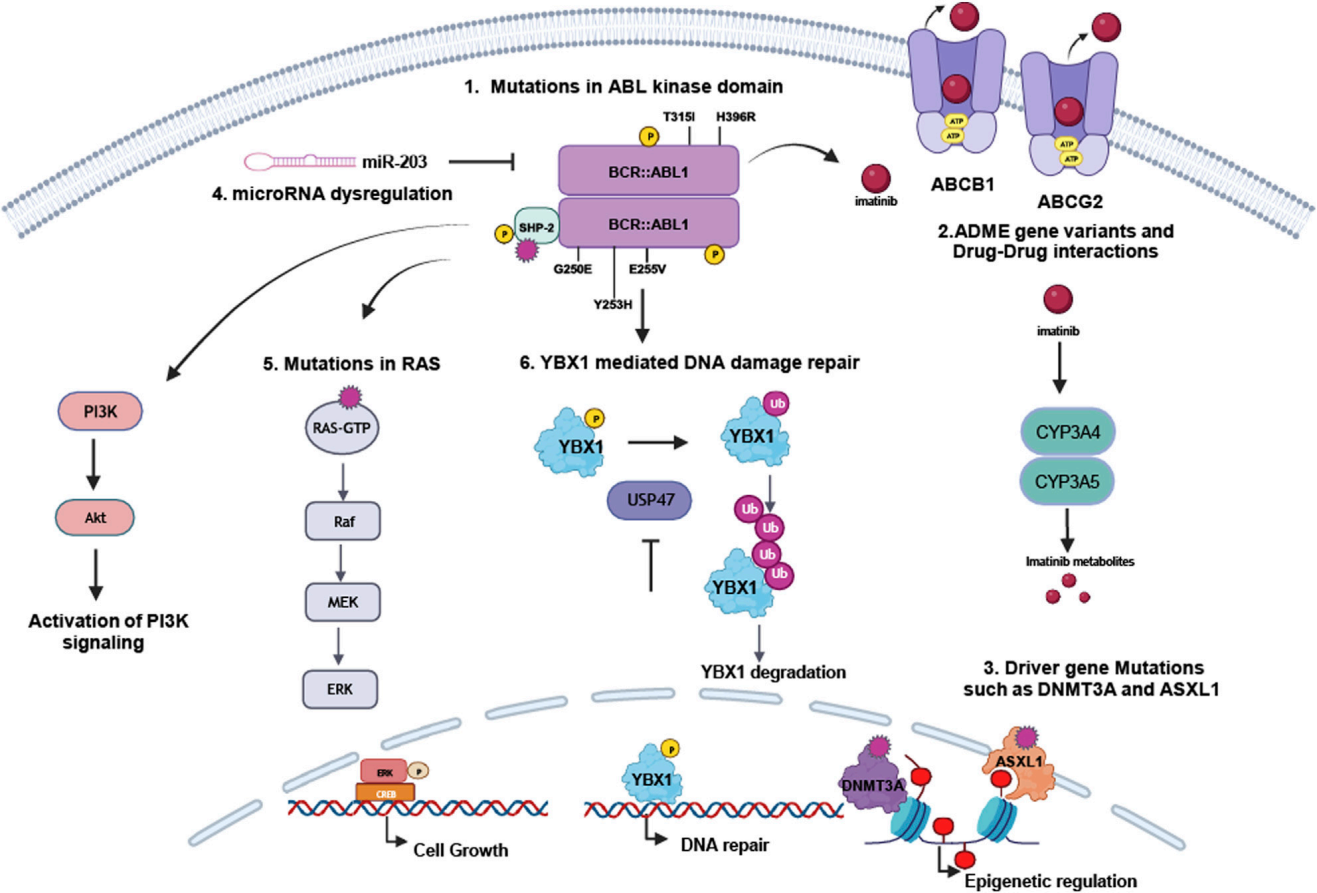
Mechanisms of resistance in *BCR::ABL1*-dependent myeloproliferative neoplasms. TKI resistance occurs either dependent or independent from the disease-causing BCR::ABL1 kinase: 1. Mutations in BCR::ABL1, especially p.G250E, p.Y253H, p.E255V, p.T315I or p.H396R, can prevent sufficient TKI binding to the kinase and by this result in therapy failure. 2. ADME genes deregulation in CYP enzymes or drug transporters, but likely also pharmacogenetic variants in these genes influence TKI metabolisms and transport. Also, drug-drug interaction interferes with TKI metabolism. TKI resistance can also be mediated by 3. driver gene deregulation or mutations, in particular in *ASXL1* or *DNMT3A*, influencing epigenetic regulation, 4. microRNA dysregulation, e.g., the *BCR::ABL1*-targeting miR-203, 5. mutations in *RAS* or *PTPN11*/SHP-2 or 6. impaired DNA repair by the YBX1-USP47-axis.

With a frequency of 12%–63%, a large subset of CML patients suffer from TKI failure due to resistance related to mutations in the BCR::ABL1 kinase or more seldomly its amplification or overexpression (Tadesse et al., 2021). Thereby, it should be noted that BCR::ABL1 mutations are more frequent in blast crisis than in chronic phase. Mutations in BCR::ABL1 often prevent sufficient binding of the TKI into the kinase domain and therefore, lead to TKI failure (von Bubnoff et al., 2002; Eiring and Deininger, 2014). The clinically most relevant mutation is the gatekeeper mutation p.T315I resulting in loss of efficacy for first and second generation TKIs, which all require a hydrogen bond formed at residue 315 to sufficiently bind to the ATP binding pocket (Reddy and Aggarwal, 2012). Options to overcome this inhibition are ponatinib, which does not require this bond due to an ethynyl group or asciminib, an allosteric inhibitor of BCR::ABL1, which binds to the kinase at the N-terminus (O'Hare et al., 2009; Hughes et al., 2019). However, does escalation of ponatinib is required to sufficiently treat p.T315I-mutated CML increasing the likelihood of cardiovascular adverse events (Braun et al., 2020). Besides p.T315I, clinically relevant BCR::ABL1 mutations are located in the P-loop, such as p.G250E or p.Y253H, in the C-lobe, e.g., p.E255V, or in the activation loop, as p.H396R, which all lead to therapy failure of imatinib, but partially also second generation TKIs (Soverini et al., 2014; Zabriskie et al., 2014; Braun et al., 2020) (Figure 2). Resistance due to BCR::ABL1 mutations can be overcome by TKI switching, as demonstrated e.g., for imatinib-resistant Y253H, which can be treated by second or third generation TKIs (de Lavallade and Kizilors, 2016). However, there is some evidence that sequential TKI therapy might lead to the development of therapy-resistant compound mutations (Shah et al., 2007; Meenakshi Sundaram et al., 2019). For these highly BCR::ABL1-mutated CMLs, combined therapy of especially ponatinib and asciminib has been discussed (Eide et al., 2019). Taken together, an in-depth analysis of the BCR::ABL1 mutational status should be evaluated in the case of TKI failure or intolerance to identify the suitable TKI for each patient (Cross et al., 2023).

### Use of pharmacogenetic variants in ADME genes as biomarkers

TKIs are predominantly substrates of CYP3A4 and CYP3A5 (Haouala et al., 2011), members of the cytochrome P450 family, leading to their inactivation and reduction in TKI plasma concentration prompting resistance as shown for imatinib and nilotinib (Peng et al., 2005). Thus, TKIs interfere with other drugs that are either substrates of CYP3A4, e.g., cyclosporin A or ketoconazole or inducers of the pregnane X receptor (PXR), which itself induces CYP3A4 and 3A5 expression, e.g., St John’s wort, carbamazepine or rifampicin (Sudsakorn et al., 2020). In addition to drug-drug interactions, pharmacogenetic variants might also influence CYP enzyme activity. While there are several variants with known attenuative effect on protein function, e.g., CYP3A4*20 (rs67666821), *22 (rs35599367) (Werk and Cascorbi, 2014) or CYP345*3 (rs776746), *6 (rs10264272) or *7 (rs41303343) (Kuehl et al., 2001), the impact of these variants on TKI response is still under debate, as studies reveal conflicting data (Kaehler and Cascorbi, 2023). Thus, further studies are necessary to clarify the role of CYP pharmacogenetic variants and use them as potential biomarkers (Figure 2). However, to prevent therapy failure by drug-drug interactions, CML patients should be informed to avoid potential detrimental drug-drug combinations.

Besides CYP enzymes, drug transporters are pivotal for the import and efflux of TKIs through barriers, but also into the CML cells. Efflux transporters of the ATP binding cassette (ABC) transporter family, especially ABCB1/P-gp (P-glycoprotein) and ABCG2/BCRP (breast cancer resistance protein), as well as importers as OCT1 (organic cation transporter 1) are known to be involved in the transport of xenobiotics into and out of the cells (Figure 2). Being deregulated in several cancers, all of these transporters were associated with anti-cancer drug resistance (Mohammad et al., 2018; Duan et al., 2023).

OCT1 is discussed to be the main protein involved in the import of TKIs. However, studies show a conflicting role in CML, as upregulation of OCT1, as well as an absence of OCT1 expression in imatinib and nilotinib resistance, but also other BCR::ABL1 TKIs was demonstrated (White et al., 2007; Nies et al., 2014; Kumar et al., 2022). In a recent study, the promoter SNP rs460089 in the organic cation transporter *OCTN1* was identified as an independent predictor of treatment-free remission under TKI discontinuation implying a role of OCTN1 in TKI response (Machova Polakova et al., 2024). Regarding TKI efflux, ABCB1 and ABCG2 were shown to be the involved in the transport of BCR::ABL1 TKIs except bosutinib, however, their contribution to TKI resistance is controversially discussed (Kumar et al., 2022). While some studies showed a transport of imatinib or nilotinib by ABCB1 (Eadie et al., 2014), others demonstrated ABCG2 as the main TKI transport protein (de Lima et al., 2014). These controversial findings might be a result of a dynamic, dose-dependent ABC transporter expression in TKI resistance. As the role of these protein itself is under debate, also the role of pharmacogenetic variants in these proteins is still widely discussed (reviewed in (Kaehler and Cascorbi, 2023)). Overall, neither the expression of ABC transporters or OCT1 nor the presence of pharmacogenetic variants in these genes can be used as a biomarker for TKI resistance in CML yet.

### Potential biomarkers of BCR::ABL1-independent TKI resistance in CML

The inhibition of the BCR::ABL1 kinase by TKIs can also be circumvented by the activation of alternative signaling pathways. Activation by external stimuli, deregulation of gene expression or mutations of the respective signaling proteins can often be found in MAP kinase, PI3K/Akt, JAK/STAT or WNT signaling pathways leading to restored proliferation, decreased apoptosis and altered cell motility and adhesion of the CML cells (Minciacchi et al., 2021) (Figure 2). Mutations in additional sex combs-like 1 (*ASXL1*), DNA methyltransferase 3 alpha (*DMNT3A*), isocitrate dehydrogenase (*IDH*) or SET binding protein 1 (*SETBP1*) were shown to be associated with CML progression and TKI resistance (Kim et al., 2017). While their effect in CML is still under debate, the presence of these mutations might be useful as biomarker for TKI resistance. However, the clinical use to optimize therapy strategies is still elusive.

*ASXL1* and *DMT3A* encode epigenetic modulators suggesting an influence of epigenetics in TKI resistance. Nevertheless, the general influence of DNA methylation or histone modifications in TKI resistance is still elusive, as there are conflicting studies (Amabile et al., 2015; Angus et al., 2018). In addition, posttranscriptional gene regulation by microRNAs, 19–21 nt short ribonucleotides facilitating mRNA decay (Krutzfeldt et al., 2006), might also be involved in the development of TKI resistance and could therefore be used as potential biomarkers. Especially the *BCR::ABL1* mRNA targeting miR-203 and miR-30a/e were shown to likely be involved in TKI response (Liu et al., 2013; Shibuta et al., 2013; Hershkovitz-Rokah et al., 2014), but also miR-144/451 and miR-150 both targeting MYC might be used as potential biomarkers of TKI resistance (Liu et al., 2012; Srutova et al., 2018). In addition, miR-142-5p was shown to be downregulated in CML patients at diagnosis, which later suffer from TKI failure and could also be used as a predictive biomarker (Klumper et al., 2020). However, prior use of these microRNAs as biomarkers, further studies are required to analyze their role in TKI resistance.

Besides these changes, our own findings in an in vitro-CML TKI resistance model demonstrate the occurrence of mutations in *NRAS*, *KRAS* and *PTPN11* in imatinib resistance (Kaehler et al., 2023). As these genes encoded oncogenes being involved in downstream signaling, they are likely associated with TKI resistance. This indicates that TKI-resistant CML patients should be analyzed on the presence of downstream mutations in the clinical routine. In addition, the role of the deubiquitinating ubiquitin-specific peptidase (USPs) in TKI-resistant CML was analyzed. In this study, the nucleic acid-binding protein Y-Box binding protein 1 (YBX1) was identified as a binding partner and substrate of the ubiquitin-specific peptidase 47 (USP47, thus, inhibition of USP47 destabilizes the YBX1 protein indicating YBX1 as a protein involved in DNA repair (Figure 2). Subsequently, loss of YBX1 via USP47 inhibition was shown to result in CML cell cycle arrest and apoptosis (Lei et al., 2021) proposing that these proteins could be used as targets to overcome TKIs resistance in CML.

### *BCR::ABL1*-negative myeloproliferative neoplasms (MPNs)

Identification of the *BCR::ABL1* transcript in CML entailed the discovery of molecular alterations as point mutations, fusion proteins and splice variants in other *BCR::ABL1*-negative leukemias. Among different MPNs, MF has a prevalence of 16,000 cases per year, while PV and ET have a higher prevalence of 160,000 cases and CNL, CEL and JMML are very rare leukemias with low incidence (Mehta et al., 2014). The main diagnostic criteria for PV are the increase of hematocrit; elevated platelets for ET, and megakaryocyte atypia and bone marrow fibrosis for PMF. However, MPNs often represent a continuum of overlapping hematological parameters with many patients experiences a transition in their disease, e.g., from ET to PV or from ET to PV to PMF (Tefferi et al., 2014; Tefferi and Pardanani, 2015; Shahin et al., 2021).

**FIGURE 3:**
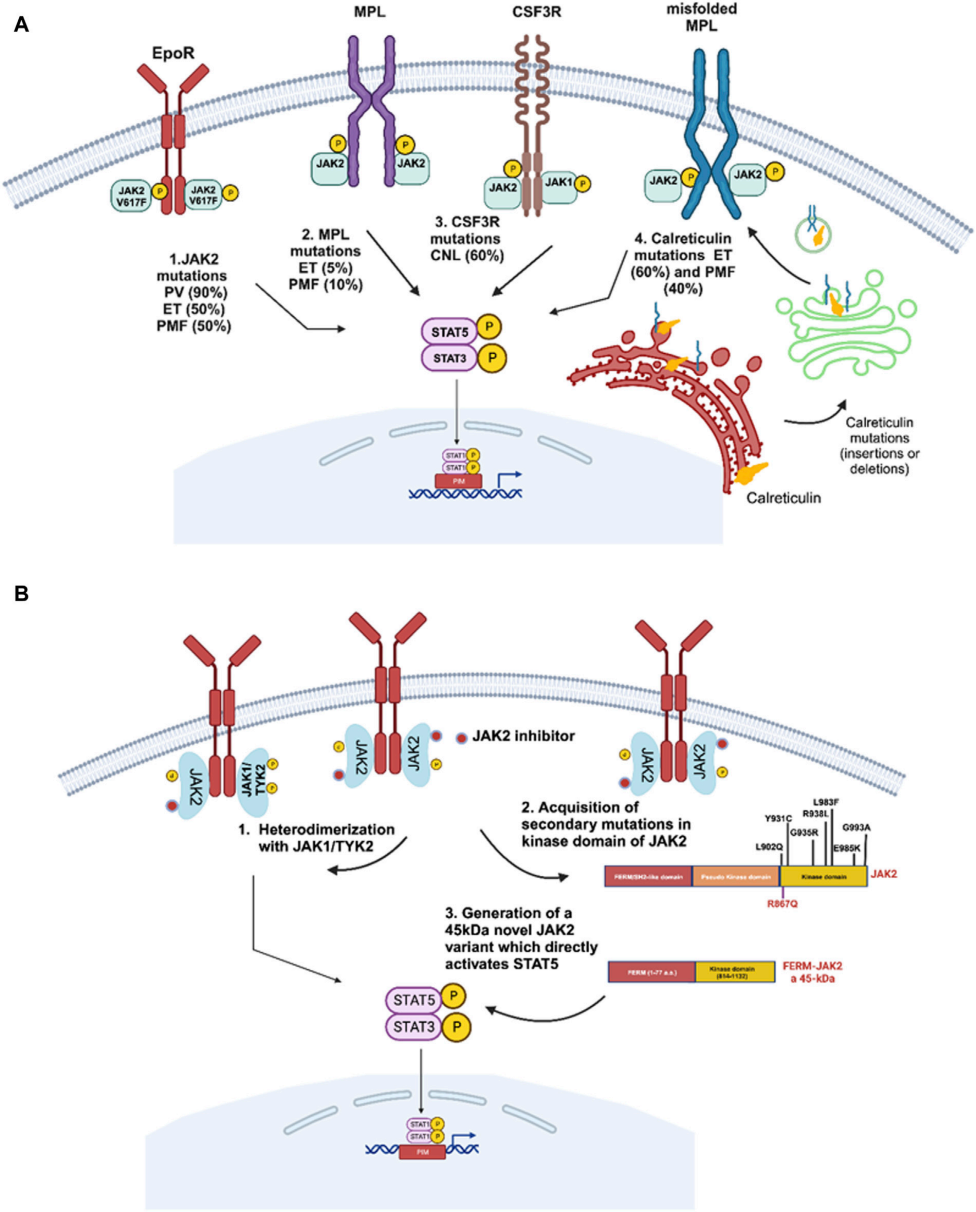
Driver gene mutations in *BCR::ABL1*-negative myeloproliferative neoplasms. **(A)** Several driver mutations have been reported in myeloproliferative neoplasms including JAK2-p.V617F in polycythemia vera (PV), essential thrombocythemia (ET) and primary myelofibrosis (PMF). *MPL* mutations are frequently associated with ET or PMF. *CSF3R* mutations are frequently reported in chronic neutrophilic leukemia (CNL), and calreticulin mutations are frequently associated with ET and PMF patients negative for JAK2-p.V617F mutation. All these alterations lead to activation and upregulation of the JAK2/STAT5 signaling pathway. **(B)** Different JAK2-dependent drug resistance mechanisms potentially involved in JAK2-inhibitor resistance resulting in sustained JAK/STAT signaling. 1. Koppikar *et al.* highlight the importance of heterodimerization of JAK2 with either JAK1 or TYK2 to reactivate JAK2 signaling (Koppikar et al., 2012). 2. The secondary resistant JAK2 mutations were demonstrated to be drivers of resistance towards JAK2 inhibitors (Deshpande et al., 2012; Kesarwani et al., 2015). 3. A short version of JAK2 was shown to provide resistance against ruxolitinib by deletion of a major part of FERM domain, SH2-like domain and pseudo kinase domain generating a JAK2 fusion with a molecular weight of 45 kDa providing strong resistance towards JAK2 TKIs (Gorantla et al., 2024).

The majority of MPNs harbor activating mutations in the Janus Kinase (*JAK2*) (Baxter et al., 2005; James et al., 2005; Kralovics et al., 2005; Levine et al., 2005), Thrombopoietin Receptor (*MPL*) (Pikman et al., 2006), Calreticulin (*CALR*) (Nangalia et al., 2013) and Colony Stimulating Factor 3 Receptor (*CSF3R*) (Maxson et al., 2013) - all resulting in constitutive JAK/STAT signaling pathway activation (Figures 1, 3A). The mutation JAK2-p.V617F is present in 90% of PV patients and 50%–60% of ET and PMF patients. PV patients lacking the JAK2-p.V617F mutation often display activating mutations in exon 12 or in 1% and 5% of the cases in MPL patients, respectively (Pikman et al., 2006; Scott et al., 2007). Besides JAK2 mutations, the majority of ET and MF patients display alterations in an endoplasmic reticulum chaperone calreticulin (*CALR*). More than 50 different insertions or deletion (indels) in *CALR* exons have been identified, with a 52 bp-deletion (type 1) and a 5 bp-insertion (type 2 mutation) being the most common (Klampfl et al., 2013; Rumi et al., 2014a; Rumi et al., 2014b). Due to these indels, a novel C-terminus in the mutant protein is generated by replacing negatively charged amino acids to neutral and positively charged ones. The 52 bp-deletion eliminates the majority of negatively charged amino acids, whereas the 5 bp-insertion retains approximately half of the negatively charged ones. Under physiological conditions, thrombopoietin receptor (*MPL*) requires calreticulin for its cell surface expression, however, due to indels in the calreticulin protein proper MPL folding is prevented resulting in cell surface expression of calreticulin along with the misfolded MPL (Elf et al., 2016). In addition to these alterations, *CSF3R* is most frequently mutated in CNL, as approximately 60% of CNL patients harbor mutations either in the membrane proximal domain or a truncation of the C-terminus (Maxson et al., 2013). All these altered proteins lead to activation or upregulation of JAK/STAT signaling pathway (Figure 3B). These molecular alterations activate JAK2 not only in cell lines, expression of these variants in *JAK2*, *MPL*, calreticulin and *CSF3R* in mice also leads to a MPN phenotype suggesting these mutations are potent drivers of MPNs (Jacquelin et al., 2020).

### Targeting JAK2 in MPNs

The tremendous success of imatinib in CML promoted targeting of JAK2 in MPNs. Several JAK family kinase inhibitors are developed and are currently tested in preclinical and clinical studies (Sonbol et al., 2013). Among those, ruxolitinib and fedratinib have been approved for treatment of intermediate and high-risk myelofibrosis, while ruxolitinib was also approved for PV patients intolerant to hydroxyurea. Unlike imatinib in CML, where already 6 months of TKI treatment can result in durable clinical response by reduction of the *BCR::ABL1* transcript, JAK2 inhibitor short-term treatment does not induce a significant reduction in MPN-driving allele burden (Verstovsek et al., 2010; Pardanani et al., 2015). Nevertheless, long-term studies on ruxolitinib indicated a reduction of the mutant allele burden, improvement of bone marrow fibrosis and increase in overall survival (Verstovsek et al., 2017a; Verstovsek et al., 2017b; Kvasnicka et al., 2018; Bose and Verstovsek, 2020). Due to these benefits, ruxolitinib remains as mainstay for the treatment of MPN patients. However, it becomes evident from clinical trials that JAK2 inhibitor treatment has limited effect on disease-driving stem cells and thus, it is unlikely that these inhibitors induce complete remission in MPN patients (Pandey et al., 2022). Below, we review potential biomarkers of JAK2 inhibitor resistance from the findings of in vitro-MPN cell lines and mouse models, as well as MPN patient data from clinical settings.

### JAK2-dependent drug resistance in MPNs

In the case of *BCR::ABL1*-mediated CML, it is clear that acquisition of secondary mutations in ABL kinase domain are one of the most prevalent drug resistance mechanism (Figure 2). However, in the case of JAK2-mediated MPNs, resistance is poorly understood on the molecular level. Several studies using genetic screens against JAK2 enzymatic inhibitors in MPN cell lines proposed a spectrum of secondary mutations, however, such mutations have not been reported in MPN patients yet (Deshpande et al., 2012; Kesarwani et al., 2015). In a study from Kopiikar *et a*l. on JAK2 resistance in MPN, ruxolitinib-resistant clones were developed being resistant to high concentration of the drug (Koppikar et al., 2012). Interestingly, these resistant clones did not display any point mutations in JAK family kinases, but heterodimeric complexes of JAK2 with other JAK family members, such as JAK1 or TYK2 in the presence of ruxolitinib (Koppikar et al., 2012) (Figure 3B). However, the functional role of heterodimerization in ruxolitinib resistance needs to be further clarified, as high dose-ruxolitinib is able to inhibit both, JAK1 and TYK2. In the same study, it was shown that drug resistance was reversible, as after ruxolitinib removal resistant clones regained their drug sensitivity suggesting the existence of non-genetic mechanisms to play a role in ruxolitinib resistance. Further, stabilization of the JAK2 protein and increase in *JAK2* expression were suggested as potential mechanism involved in ruxolitinib resistance (Koppikar et al., 2012). In line with this study, Tvorogov *et al.* suggested that accumulation of JAK-activation loop phosphorylation was linked to type I JAK inhibitor withdrawal syndrome in myelofibrosis (Tvorogov et al., 2018). This highlights that ruxolitinib induces structural changes in the activation loop conformation, which in turn affect the JAK2 protein level and activity contributing to JAK2 inhibitor persistence in MPN patients. In a previous study from our own group, we also clearly demonstrated that ruxolitinib preferentially binds to active JAK2, when the activation loop is fully phosphorylated and this binding stabilizes the activation loop conformation inside the kinase domain, which itself restricts the assessment to phosphatases to the activation loop tyrosines 1007/1008. When the drug dissociates from the fully phosphorylated JAK2, this leads to hyperactivation of downstream signaling (unpublished data, Figure 3B). This data suggests that identification of crucial pathway responsible for withdrawal syndrome is important in order to increase the efficacy of ruxolitinib therapy for MPN patients.

Using a mutagenesis screening to identify drivers of ruxolitinib resistance, Deshpande *et al.* demonstrated a total seven different exchanges in the JAK2 kinase domain associated with ruxolitinib resistance (Deshpande et al., 2012). Among these seven variants, only two, p.Y931C and p.G935R, significantly induced resistance (Figure 3B). Interestingly, variants at these residues also confer cross resistance against other JAK2 inhibitors, such as lestauritinib, fedratinib and AZD 1480 (Deshpande et al., 2012). Interestingly, the gatekeeper residue JAK2 mutation p.M929I seems to be of minor relevance, as it does not provide a strong resistance phenotype suggesting that presence of an isoleucine at this residue does not hinder ruxolitinib binding, which is in contrast to other kinases, where e.g., introduction of a bulky methyl group containing isoleucine (p.T315I) in ABL1 sterically hinders TKI binding.

Further, in a study analyzing the functional role of the JAK2 domains, namely, FERM, SH2-like, the pseudo kinase and kinase domain, in drug resistance, ruxolitinib-resistant clones were established displaying several JAK2 variants (Kesarwani et al., 2015). However, biochemical characterization of these variants suggests only a few residues located in the kinase domain, such as p.L902Q, p.Y931C, and p.L983F, significantly affect the IC50 value (Kesarwani et al., 2015). Interestingly, the ruxolitinib-resistant mutation p.L983F is very sensitive to fedratinib suggesting a substrate specificity. In addition to point mutations in the kinase domain, in our study, we identified a novel 45 kDa JAK2 variant present at higher ruxolitinib concentrations, which is generated by deletion of parts of the FERM domain, SH2-like domain and pseudo kinase domain (Figure 3B). This 45 kDa JAK2 variant is an in-frame fusion protein consisting of the N-terminal 77 amino acids together with residues 814–1132 of the kinase domain and able to activate STAT5 independent of the cytokine receptor, to constitutively dimerize and to prevent the phosphorylation of activation loop tyrosines 1007/1008. Introduction of this variant in mice led to a lethal MPN-like phenotype and myelofibrosis (Gorantla et al., 2024). This suggests an urgent need for whole exome sequencing or panel sequencing in clinical monitoring of inhibitor-refractory individuals to detect JAK2 variants. This is stressed by data from whole exome sequencing and single cell genotyping to determine clonal evolution in myelofibrosis patients during ruxolitinib treatment (Mylonas et al., 2020). In this study, surprisingly two out of 15 patients achieved molecular remission during therapy, even though one of the patients attained the JAK2 inhibitor resistant p.R867Q mutation (Marty et al., 2014; Mylonas et al., 2020). This is one of the rare examples of an acquisition of a secondary JAK2 mutation, which to instill resistance towards JAK2 inhibitors. Taken together, aberrations in the JAK2 kinase should be clinically monitored and considered as relevant biomarkers of drug failure.

### JAK2-independent drug resistance in MPNs

**FIGURE 4:**
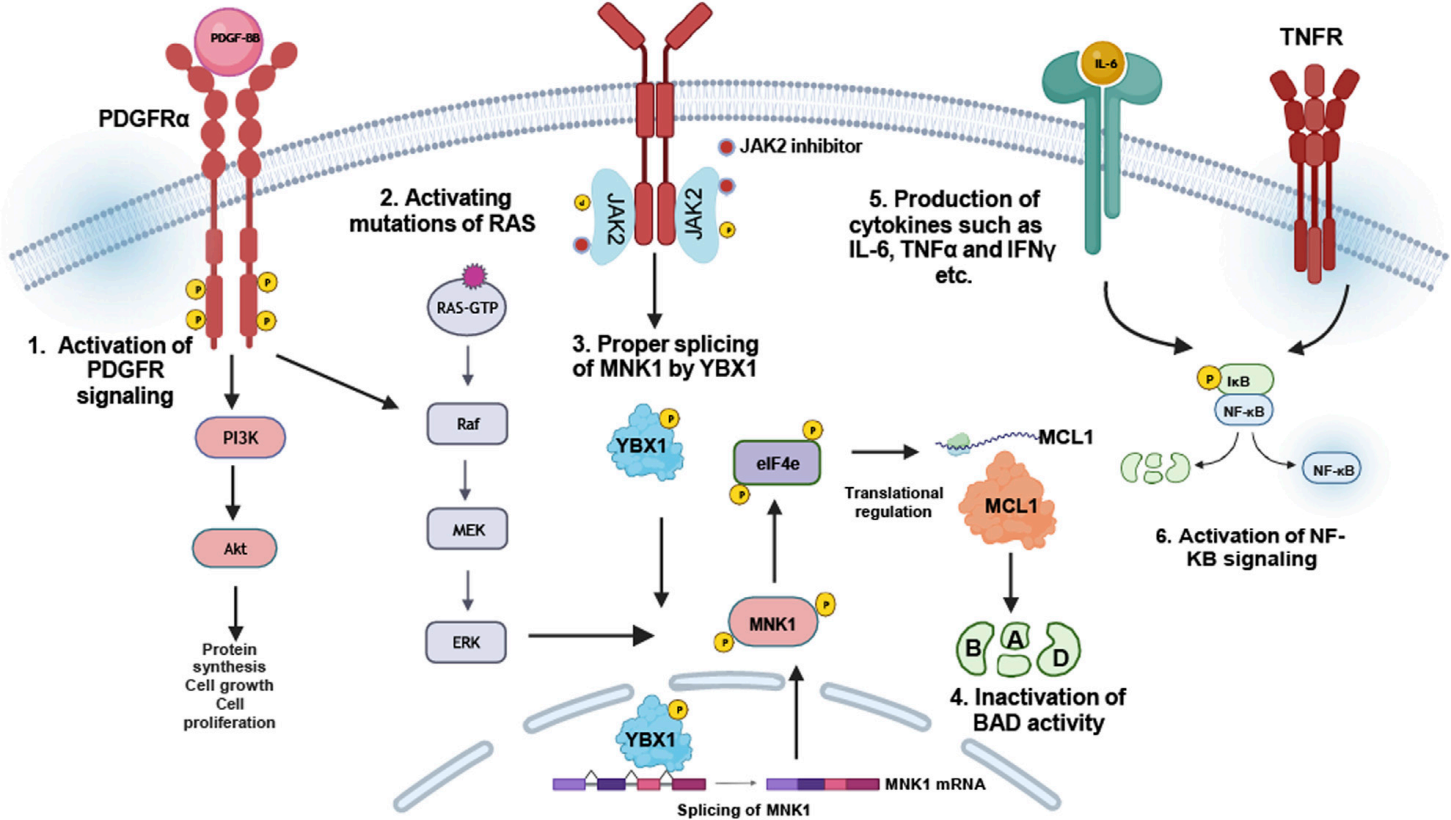
JAK2-independent resistance mechanisms: JAK2 inhibitor resistance can be provided by compensatory signaling pathway activation: 1. Upregulation of PDGFRα-signaling facilitates sustained PI3K/Akt and Ras-MAP kinase signaling (Stivala et al., 2019). 2. Activation mutations of RAS were identified in ruxolitinib-resistant myelofibrosis patients (Mylonas et al., 2020). 3. Regulation of YBX1 phosphorylation by ERK1/2 in JAK2-p.V617F cells is responsible for MNK1 splicing, which itself regulates MCL-1, required for inactivation of BAD (Jayavelu et al., 2020). 4. Upregulation or induction of certain cytokines, especially IL-6, TNFα and IFNγ, suggest the induction of NFkB pathway to be responsible for JAK2 inhibitor resistance in MPN patients (Fisher et al., 2019).

In addition to the intrinsic role of JAK2 in JAK2 inhibitor persistence, several other mechanisms were proposed to identify therapeutic vulnerabilities of JAK2 inhibitor persistence in MPNs. Using a murine MPN model, extrinsic cellular mechanisms were shown to provide a survival signal of MPN-inducing cells in the presence of JAK2 inhibitors (Stivala et al., 2019). In this study, a phospho-receptor tyrosine kinase arrays coupled with multiplexed RNA expression analysis of bone marrow cells and splenocytes of JAK2-p.V617F and MPL-p.W515L identified ERK activation even in the presence of JAK2 inhibitors. This led to the identification of PDGFRα activation by PDGF as a potential mediator of ERK activation during JAK2 inhibitor therapy. Further, it was demonstrated that both, PDGF ligands and PDGFRα were induced in megakaryocyte-erythroid progenitor cells, as well as PDGFRα expression in bone marrow of leukemic stem cells (LSCs) and common myeloid progenitor cells (CMPs) (Stivala et al., 2019). This highlights that induction of RTK-mediated signals, such as activation of PDGFRα signaling, is able to antagonize the effects of JAK2 inhibitors leading to survival of LSCs and CMPs even in the presence of ruxolitinib (Figure 4). Nevertheless, the involvement of PDGFR signaling in JAK2 inhibitor refractory MPN patients remains to be further elucidated.

Beside PDGFR, mutations in the Ras pathway genes were shown to be associated with ruxolitinib resistance in patients (Mylonas et al., 2020). In more detail, three patients transformed to leukemia associated with time-dependent occurrence of mutations in *NRAS* or *KRAS* genes (Mylonas et al., 2020). Acquisition of Ras-activating mutations contributed resistance against JAK2 inhibitors also in myelofibrosis patients (Figure 4). Therefore, identification of these mutations, even in a small subset of cells, is absolutely important and provides the potential opportunity for altered clinical management, such as combination treatment strategies, e.g., JAK2 inhibitor together with Ras pathway inhibitors, to intervene disease progression.

Using a global phosphoproteomic analysis of the JAK2 signaling landscape associated with JAK2-p.V617F, it was reported that the most enriched cellular processes associated specifically with JAK2-p.V617F signaling were RNA splicing and processing (Jayavelu et al., 2020). Using an RNA interference screen focusing on proteins involved in RNA splicing and processing, YBX1 was shown to be involved in sensitizing cells to growth inhibition and induction of apoptosis by ruxolitinib (Jayavelu et al., 2020). *YBX1* knockdown in a human JAK2-p.V617F model cell line sensitized the cells to JAK2 inhibitor treatment. Further, it was demonstrated that YBX1 was phosphorylated in JAK2-p.V617F in a MEK/ERK-dependent manner on serine residue 30 and 34 enforcing nuclear localization of YBX1 (Jayavelu et al., 2020). MEK inhibition reduced this effect suggesting that JAK2 inhibition alone may not antagonize YBX1 contributing to JAK2 inhibitor persistence. Alterations in mRNA splicing in JAK2-p.V617F cells led to identification of intron retention of several mRNAs encoding proteins involved in RNA splicing, non-sense-mediated decay or apoptosis (Jayavelu et al., 2020). One potential gene involved is the MAPK-interacting kinase (MNK1) being downregulated in JAK2-p.V617F cells after YBX1 depletion. It was shown that the YBX1 splicing function is required for the expression of MNK1, which in turn depends on ERK signaling by JAK2-p.V617F (Figure 4). Thus, inhibition of ERK signaling in JAK2 inhibitor persistent cells by impending YBX1 expression is able to deregulate MCL1 and BIM expression to favor apoptosis (Jayavelu et al., 2020). This shows a novel synthetic gene-drug lethality in JAK2-mediated MPNs. Taken together, targeting MEK/ERK or MNK1 could be beneficial in JAK2 inhibitor therapy in MPN patients to avoid persistence.

In order to identify signaling pathways inducing JAK2 inhibitor resistance in MPNs, a screen using gain-of-function mutants of signaling proteins was performed (Winter et al., 2014). Mutations of Ras, MEK and AKT were shown to antagonize JAK2 inhibition in MPN model cell lines, while combinational treatment approaches, such as the inhibition of AKT and MEK were able to reduce the IC50 of JAK2 inhibitors. This study also demonstrated that inactivating phosphorylation of BAD protein plays a key role in cell survival in response to JAK2 inhibition (Winter et al., 2014), as JAK2 signaling results in phosphorylation and inactivation of BAD via ERK, PIM as well as AKT (Fiskus et al., 2013; Bhagwat et al., 2014; Mazzacurati et al., 2019; Kuykendall et al., 2020). Overall, these studies demonstrated a compensatory signaling mechanism activating ERK, AKT and Ras leading to inactivation of BAD and subsequent prevention of apoptosis in response to JAK2 inhibition. These observations suggest that the anti-apoptotic activity of BCL-2 proteins in JAK2-p.V617F MPNs is due to inactivation of BAD. Thus, inhibition of BCL-2 family members might be beneficial for MPN patients (Figure 4). Consistent to this hypothesis, a study by Pemmaraju *et al.* demonstrated the efficacy of ruxolitinib in combination with navitoclax (a BCL-2 inhibitor) in myelofibrosis patients (Pemmaraju et al., 2020).

A study including mass cytometry analysis identified cytokine overproduction in myelofibrosis, which might also be involved in JAK2 inhibitor persistence in MPN patients (Figure 4). Using *ex vivo* thrombopoietin (TPO) stimulation followed by ruxolitinib treatment, IL-6, IL-8, IL-10 and TNFα were shown to be stimulated by TPO, yet these cytokine levels were not reduced by ruxolitinib treatment (Fisher et al., 2019). Treatment with small molecular inhibitors of NF-κB (pevonedistat) and MAP kinases (trametinib, JNKi8, and VX-745, inhibitors of MEK and JNK and p38, respectively) inhibited cytokine release. Given that ruxolitinib alone is inefficient to downregulate certain inflammatory cytokine levels, targeting the crucial signaling pathway, in particular NF-kB, might antagonize the pro-inflammatory state in MPNs and be beneficial for ruxolitinib-resistant MPN patients (Figure 4).

## Conclusion and future perspectives

*JAK2*- and *BCR::ABL1*-are oncogenes giving rise to distinct tumor entities. However, the constitutive expression or activation of these oncogenes leads to sustained proliferative signaling and tumor development. Despite all discrepancies, the resistance mechanisms in the targeted treatment of these diseases share some similarities: Mutations in the targeted oncogene often prevent sufficient TKI binding and therefore, result in therapy failure (such as in BCR::ABL1 for CML and in JAK2 for JAK2-driven PV, ET or PMF). Further, sustained signaling pathway activation by the presence of downstream mutations (e.g., *NRAS* or *ASXL1*) or deregulation of gene expression (e.g., ERK, PDGFR or BAD) can be observed in TKI resistance. USPs might also be involved in TKIs resistance in both, BCR::ABL1-positive and JAK2-p.V617F MPNs. These similarities suggest that TKI treatment in general should be accompanied by mutational screening of the targeted oncogene to identify potentially harmful mutations. Further, also exome sequencing to recognize changes in downstream signaling pathways should also be considered. As this is a challenge in the clinical daily life, the previous identification of mutations in in vitro-models can be tremendously helpful to limit the necessity of exome analyses, in order to identify single aberrations, which could be easily tested with distinct tests. In addition, the similarities between the resistance mechanisms also implies that the cancer entity itself becomes more unimportant, while the molecular properties are paramount.

### Methodology

The review is based on a literature search in PubMed applying the keywords “CML, MPN, drug resistance, tyrosine kinase inhibitors, imatinib, ruxolitinib, JAK2 and BCR::ABL1”, including some of our own contributions. The figures were designed using BioRender.
